# Supplementary material for: H3K36 Methylation as a Guardian of Epigenome Integrity
Source: Nat Commun. 2025 Dec 11;16:11371. doi: 10.1038/s41467-025-66365-9 (PMC12727757; doi:10.1038/s41467-025-66365-9)
Supplement: Supplementary file 1 — Supplementary Information [file 41467_2025_66365_MOESM1_ESM.pdf]

H3K36 Methylation as a Guardian of Epigenome Integrity

Supplementary Information

Padilla and Shipman et al.

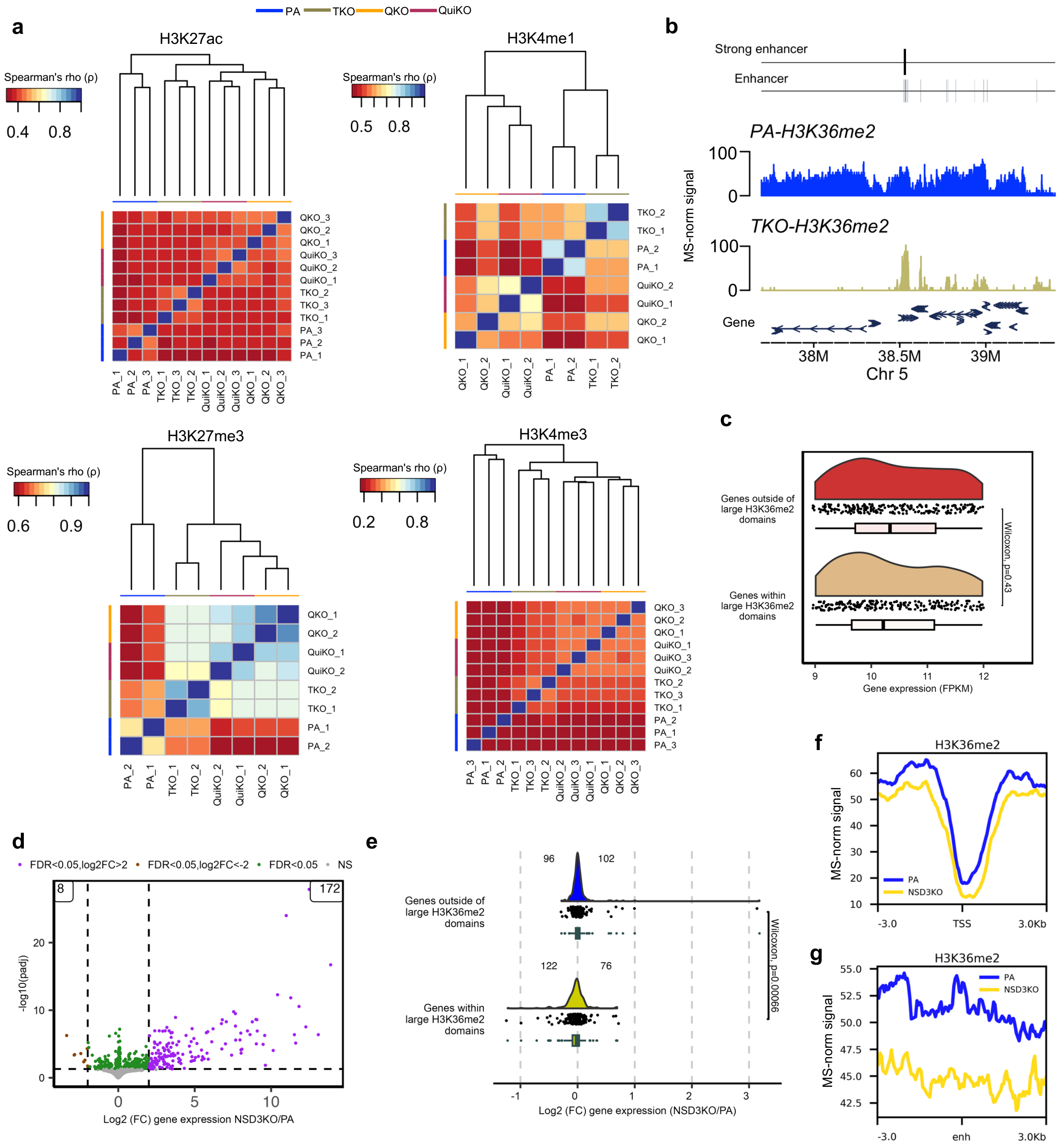

Supplementary Figure 1. Analysis of the downstream consequences of focal loss of H3K36me2. a Correlation matrix heatmaps of genome-wide H3K27ac, H3K4me1, H3K27me3 and H3K4me3 signals binned in 1 kb windows demonstrate strong reproducibility between replicates, with samples from each cell line clustering together. Spearman's rho was calculated. **b.** Representative genome-browser tracks showing

NSD1/2-SETD2-TKO leads to depletion of broad H3K36me2 domains, with the remaining, smaller H3K36me2 regions (i.e. peaks) found at clusters of enhancers. **c.** Gene expression plots showing that the two gene groups (genes outside of large H3K36me2 peaks (n=219) and genes within large H3K36me2 peaks (n=219)) have no significant difference in basal expression (FPKM) in the parental (wildtype) cell line. **d.** Volcano plot showing differential gene expression between NSD3KO and parental cell lines. **e.** Gene expression log2 fold changes of NSD3KO versus parental cells showing that in the same set of genes within the large H3K36me2 peaks from TKO, 122 genes become downregulated whereas 76 become upregulated. In a control set of genes, a similar number of genes become upregulated (96) and downregulated (102). **f.** Aggregate plot of H3K36me2 centered on the TSS of the set of genes within large H3K36me2 peaks from **e,** showing a decrease in NSD3KO compared to parental samples. **g**. Aggregate plot of H3K36me2 signal centered on annotated enhancers closest to the genes from **f**, indicating a stronger decrease at the enhancers of these target genes. For **b, f** and **g**, MS-normalized signals were computed as previously described in Fig. 1 and represent the mean local frequency of the relevant modification. In **c** and **e**, a two-sided Wilcoxon rank-sum test was performed. Source data are provided as a Source Data file.

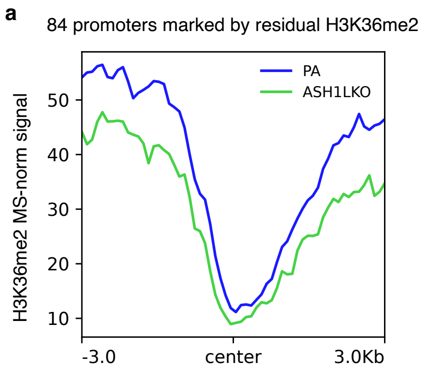

Supplementary Figure 2. KO of ASH1L independently does not lead to significant depletion of H3K36me2. a Aggregate plot of MS-normalized H3K36me2 signal centered on the 84 promoters marked with residual H3K36me2 in the QKO cells, showing similar H3K36me2 levels between PA and ASH1L-KO at these promoters (n=84).

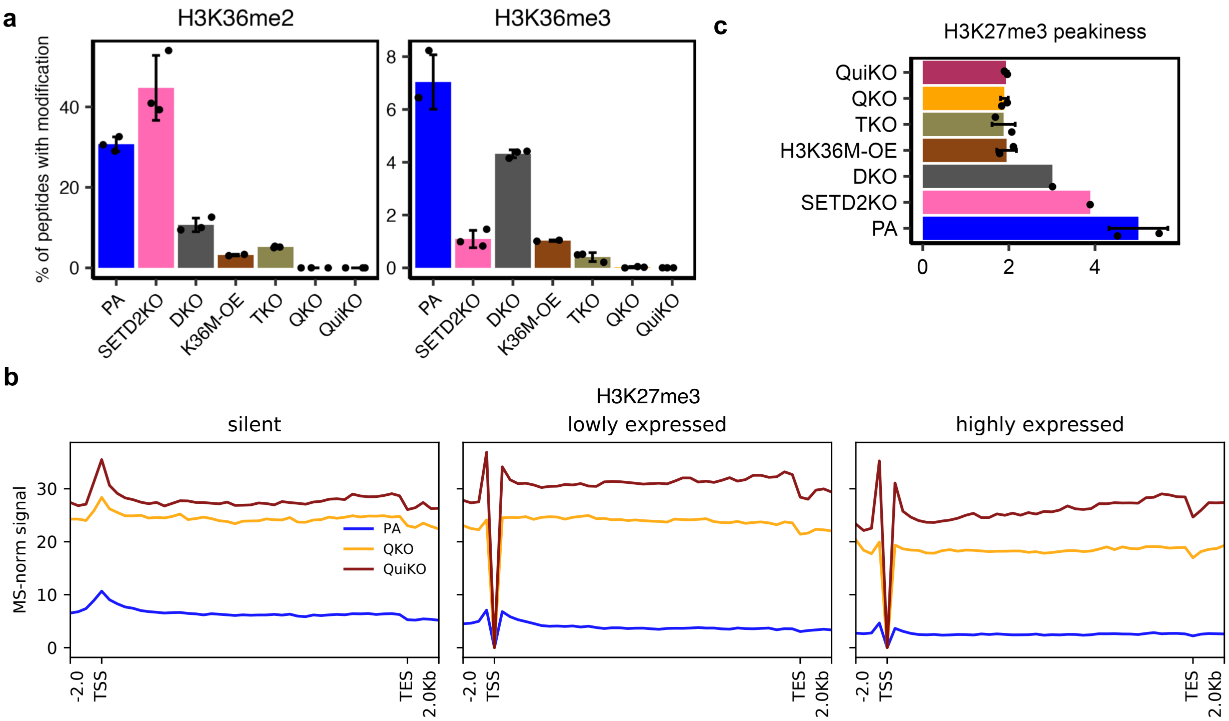

Supplementary Figure 3. Analysis of H3K27me3 broadening following H3K36me depletion. a Barplots of genome-wide prevalence of modifications based on mass spectrometry showing global loss of H3K36me2/3 following knockouts of K36MTs. **b** Metagene plots of MS-normalized H3K27me3 CUT & RUN signal plotted within the same sets of genes from Fig.3a, showing increasing invasion of H3K27me3 into gene bodies with further depletion of H3K36me2 in QKO and QuiKO. **c** H3K27me3 “peakiness” plot showing decreased “peakiness” scores, indicating broadening of H3K27me3 (broader distribution) following multiple knockouts of K36MTs. For **a** and **c**, error bars display the standard deviation around the mean. (n=3 per condition). Source data are provided as a Source Data file.

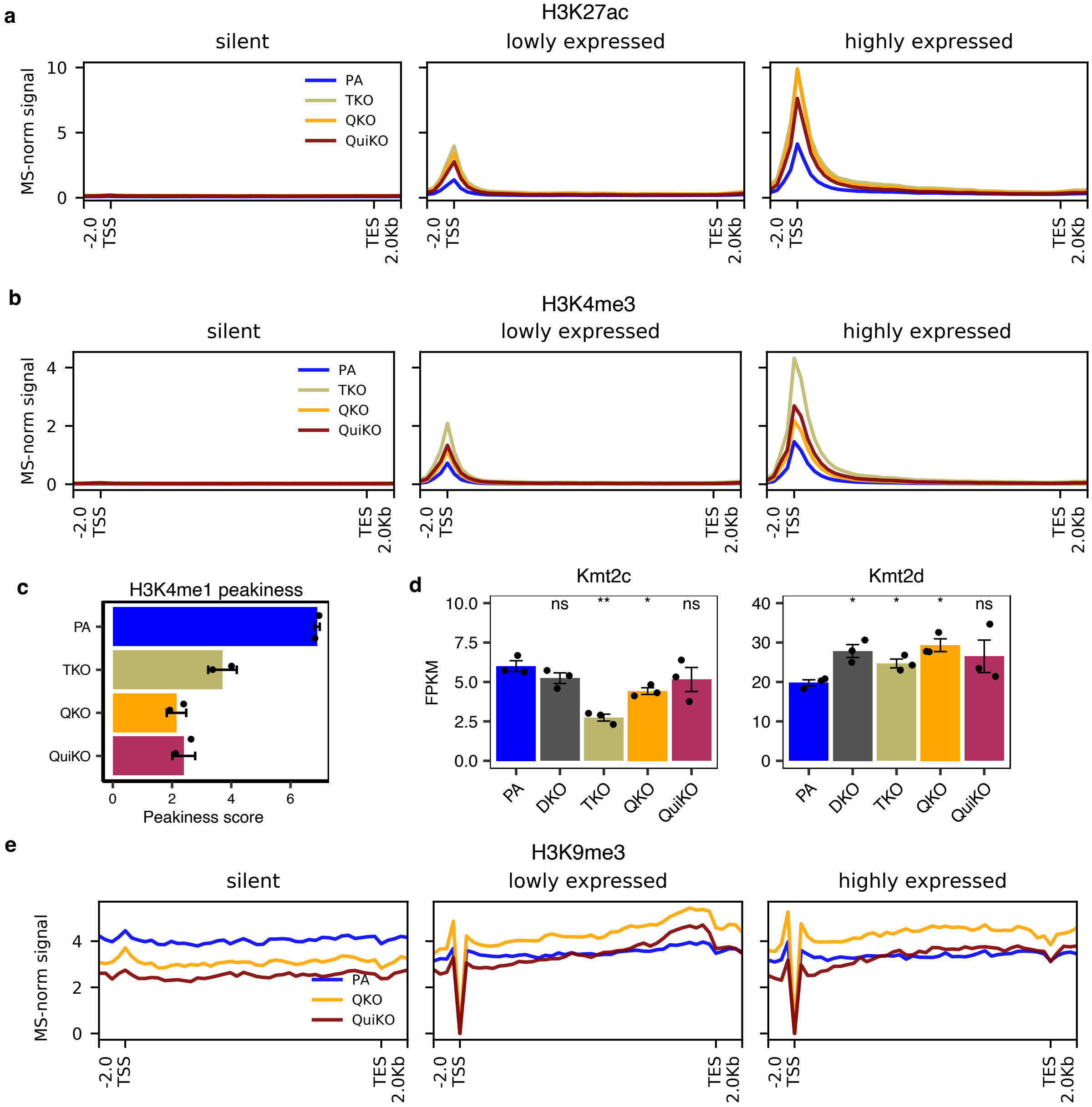

Supplementary Figure 4. Analysis of other histone marks within gene bodies. a-b Gene-body metaplots of MS-normalized H3K27ac and H3K4me3 ChIP-seq signals respectively, illustrating lack of changes within genes (from the same groups of genes in Fig. 3). **c** Barplot of H3K4me1 peakiness scores, showing decreased peakiness in the multi-KOs, which is indicative of less focused H3K4me1 distributions. error bars display the standard deviation around the mean. (n=2 per condition) **d** Barplots showing significant downregulation of KMT2C in TKO and QKO cells whereas significant upregulation of KMT2D occurs in DKO, TKO and QKO cells. Error bars display the

standard deviation around the mean. (n=3 per condition). *P*-values derived from a two-sided student's *t*-test comparing individual conditions against the PA sample and adjusted using the Benjamini–Hochberg (BH) procedure. ns > 0.05, * < 0.05 and ** < 0.01. Adjusted *p*-values for KMT2C are: DKO = 0.244; TKO = 0.009; QKO = 0.045; QuiKO = 0.388. Adjusted *p*-values for KMT2D are: DKO = 0.036; TKO = 0.036; QKO = 0.036; QuiKO = 0.242. Gene expression levels were FPKM (fragments per kilobase of transcript per million mapped reads) normalized. (n=3 per condition). **e** Gene-body metaplots of MS-normalized H3K9me3 CUT & RUN signal, showing increased H3K9me3 within the gene bodies of lowly and highly expressed genes for QKO compared to parental (PA) cells. Source data are provided as a Source Data file.

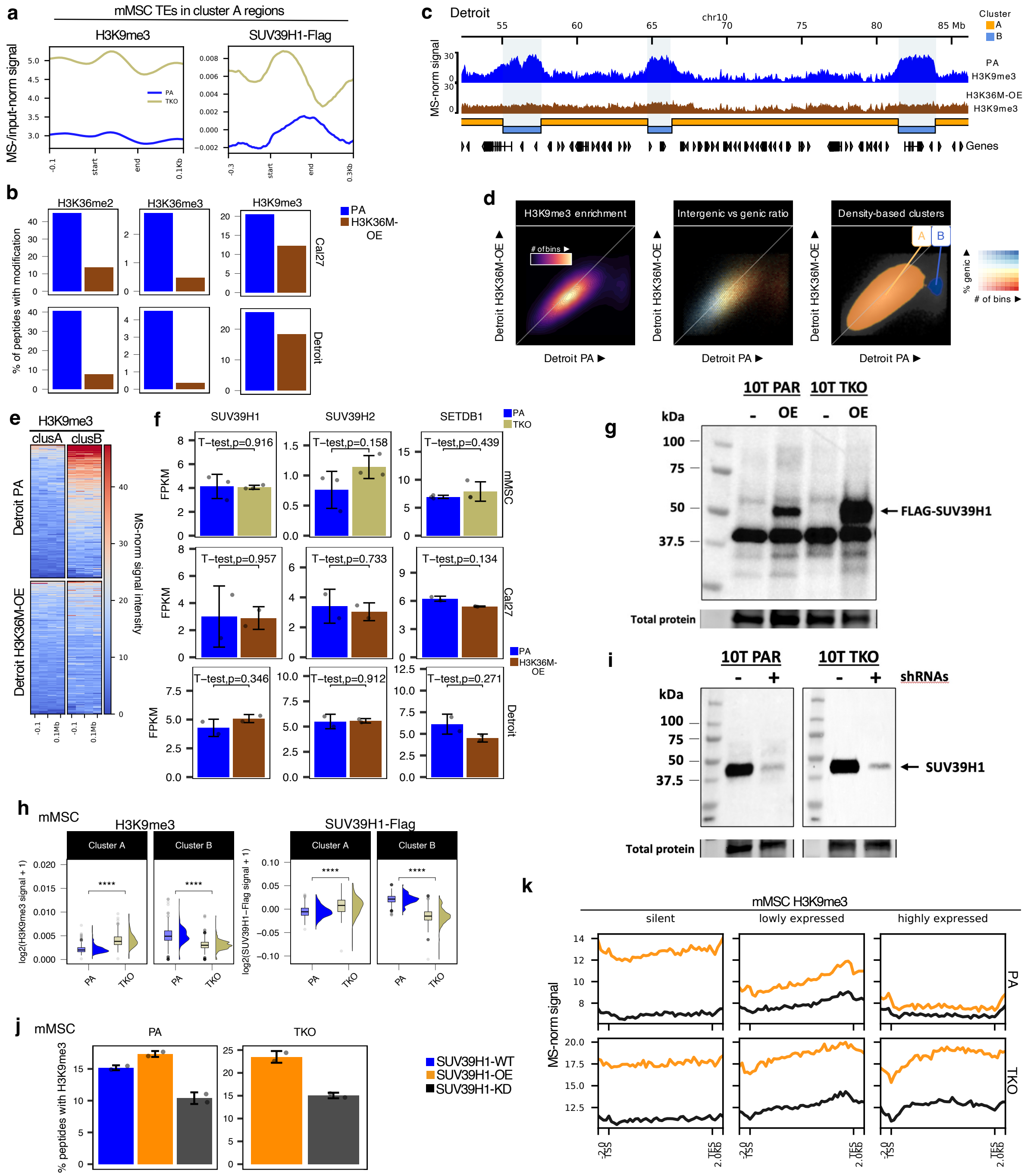

Supplementary Figure 5. Changes in H3K9me3 levels following H3K36me depletion across cell lines. a Aggregate plots of H3K9me3 and SUV39H1-FLAG enrichment centered on transposable elements show elevated signals in TKO mMSCs. **b** Barplots of showing a global decrease in H3K36me2/3 and H3K9me3 in H3K36M-OE HNSCC cells compared to their respective parental controls. **c** Genome browser tracks illustrating loss

of H3K9me3 domains in H3K36M-OE Detroit562 cells. **d** Genome-wide scatterplots of 10-kb bins, demonstrating reduced intergenic H3K9me3 (Cluster B) in H3K36M-OE Detroit562 cells. **e** Heatmaps showing decreased H3K9me3 signal in cluster B regions in H3K36M-OE Detroit562. **f** Barplots of SUV39H1, SUV39H2 and SETDB1 FPKM levels indicating no significant differences comparing H3K36me-deficient cells to their respective parental controls. *P*-values derived from a two-sided student's *t*-test. (n=3 for mMSC and n=2 for HNSCC) **g** Western blot confirming FLAG-SUV39H1 overexpression in parental and TKO mMSCs. **h** Box- and violin-plots showing increased H3K9me3 and SUV39H1-Flag signals in cluster A and decreased in cluster B following TKO in mMSCs. Two-sided Wilcoxon rank-sum test was performed followed by Benjamini-Hochberg correction. For H3K9me3, adjusted *p*-values were: cluster A = 2.9e-54, n = 708 bins; cluster B = 4.3e-185, n = 2408. For SUV39H1-Flag: cluster A = 5.6e-16, n = 708; cluster B = 1.4e-186, n = 2390. **i** Western blot confirming SUV39H1 knockdown in parental and TKO mMSCs. **j** Barplots of H3K9me3 genome-wide abundance, showing increased abundance following SUV39H1-OE and decreased abundance following SUV39H1 knockdown. (n=2) **k** Aggregate plots centered on gene-bodies, stratified by gene expression, reveal that in parental cells, SUV39H1-OE increased enrichment primarily at silent genes. In contrast, in TKO, SUV39H1-OE leads to elevated enrichment across silent and actively expressed genes. For **a, c, e, h,** and **k,** ChIP-seq signals were either normalized by MS (H3K9me3) or by input (SUV39H1-FLAG). For the boxplots in **h**, boxes span the first and third quartile, median is indicated with a center line and whiskers extend to 1.5 times the interquartile range. For the barplots in **f** and **j,** error bars display the standard deviation around the mean. Source data are provided as a Source Data file.

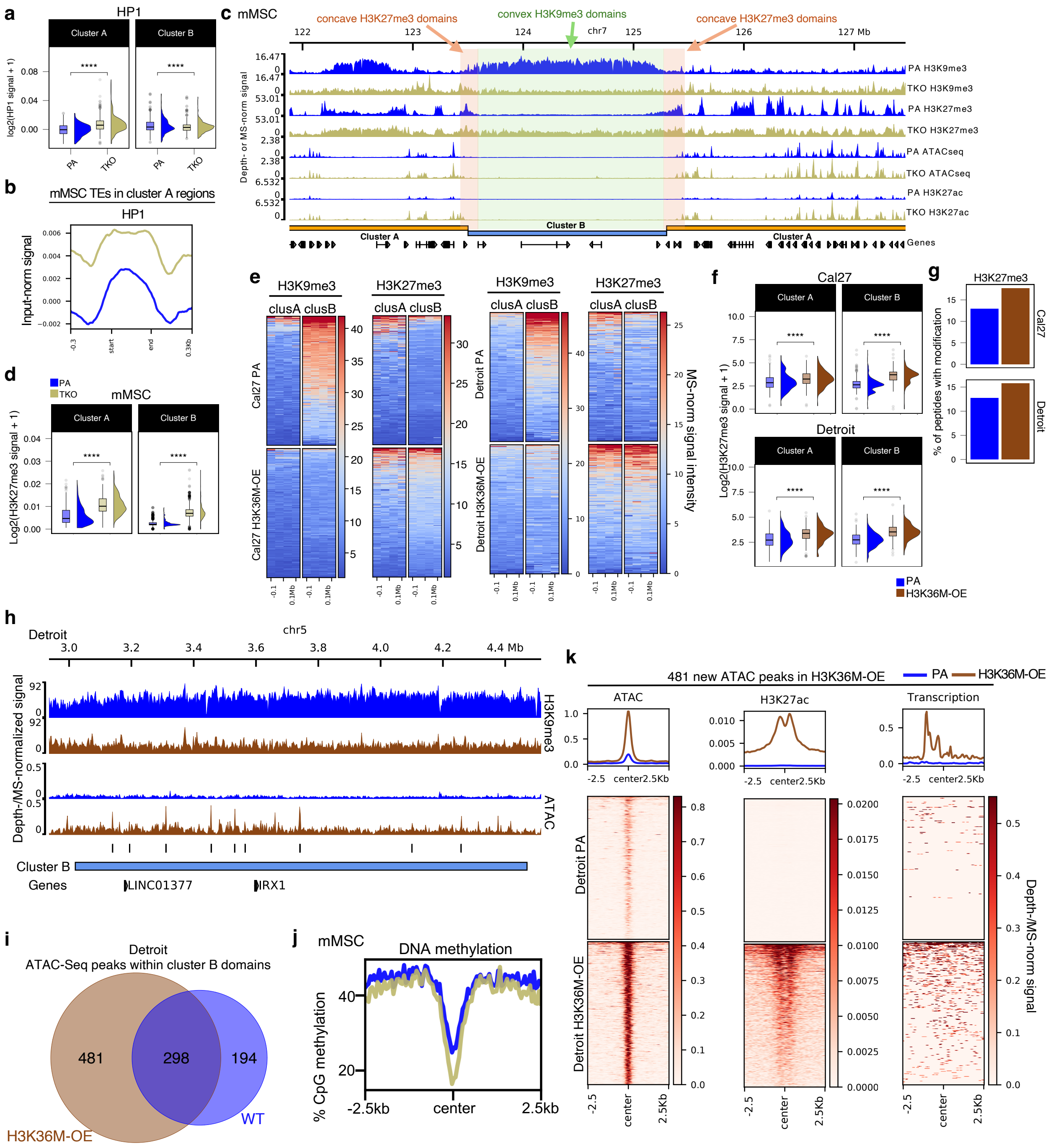

Supplementary Figure 6. Downstream epigenetic cascade following H3K9me3 loss is recapitulated in HNSCC. a Box and violin plots of input-normalized signals show that HP1 signal is significantly increased in cluster A and decreased in cluster B in TKO. *P*-values: Cluster A = 5.6e-26, n = 708 bins; Cluster B = 3.5e-09, n = 2390. **b** Aggregate plot of HP1 signal centered on TEs, showing increased enrichment in cluster A regions in TKO mMSCs. **c** Genome browser tracks showing H3K9me3 loss and H3K27me3 gain

upon H3K36me depletion in TKO mMSCs. **d** Box- and violin-plots showing H3K27me3 increase in clusters A and B regions in TKO mMSCs. *P*-values: Cluster A = 8.1e-51, n = 708 bins; Cluster B = 5.7e-198, n = 2408. **e** Heatmaps showing reduced H3K9me3 signal in cluster B regions and increased H3K27me3 signal in both clusters in H3K36M-OE HNSCC cells. **f** Box and violin plots indicating a significant increase of H3K27me3 in both clusters comparing H3K36M-OE to parental cells in HNSCC. Cal27 *p*-values: cluster A = 2.9e-34, n = 560; cluster B = 2.7e-44, n = 524. Detroit562 *p*-values: cluster A = 1.2e-29, n = 390; cluster B = 1.8e-33, n = 414. **g** Barplots illustrating a global H3K27me3 increase in H3K36M-OE. **h** Genome browser tracks depicting newly accessible regions within cluster B regions in Detroit562 H3K36M-OE. **i** Venn diagram comparing the number of ATAC-seq peaks found in cluster B regions, where new peaks appear in H3K36M-OE Detroit562. **j** Aggregate plot of DNA methylation centered on the 1914 ATAC-Seq peaks opening in mMSC TKO, indicating a downregulation of CpG methylation. **k** Heatmaps centered on 481 new ATAC-Seq peaks in H3K36M-OE Detroit562, showing upregulation of H3K27ac and increased transcriptional activity. **** adjusted *p*-value < 0.0001 from a two-sided Wilcoxon rank-sum test followed by BH correction. For **a, d** and **f,** boxes span the first and third quartile, median is indicated with a center line and whiskers extend to 1.5 times the interquartile range. Normalized signals were either depth-normalized (ATAC-Seq), MS-normalized or RPKM (reads per kilobase per million mapped) transformed. Source data are provided as a Source Data file.

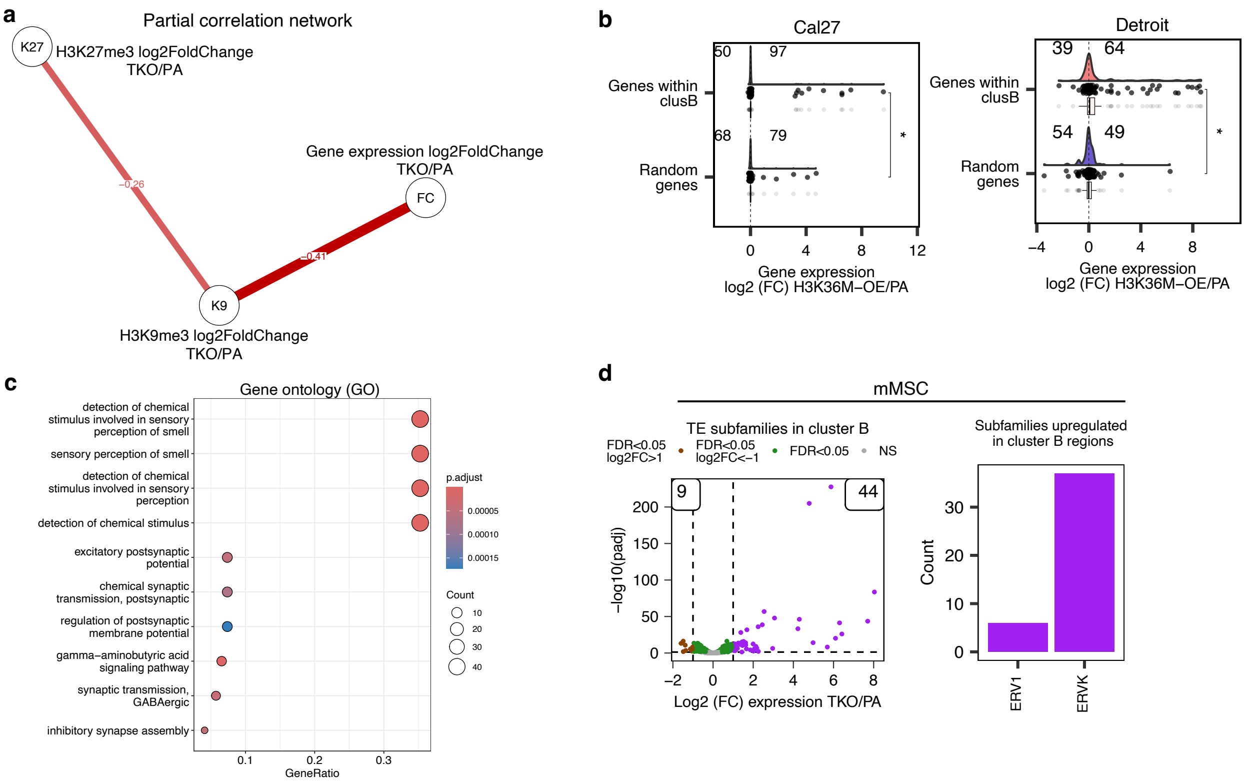

Supplementary Figure 7. Ectopic H3K27me3 accumulation is insufficient to maintain transcriptional silencing in regions of H3K9me3 loss. a Partial correlation analysis comparing TKO to parental mMSCs indicates that H3K9me3 fold changes are strongly correlated with gene expression changes in cluster B regions. There is a moderate negative correlation between H3K27me3 and H3K9me3 fold changes in cluster B regions, whereas no correlation exists between H3K27me3 and gene expression changes. n = 260 genes in cluster B regions used. **b** Log2 fold gene expression changes comparing H3K36M-OE to their respective parental controls (Cal27 and Detroit562), showing genes in cluster B regions are more upregulated than downregulated (97 versus 50 and 64 versus 39 in Cal27 and Detroit respectively) compared to a randomized, control set of genes, which have similar numbers of genes up- and downregulated (79 versus 68 in Cal27 and 49 versus 54 in Detroit562). Cal27 *p*-value = 0.024; Detroit562 *p*-value = 0.044. **c** Over-representation analysis of gene ontology (GO) terms for genes common to both mMSCs and Cal27 losing H3K9me3—following TKO and H3K36M-OE, respectively; Olfactory and GABA-related terms are among the top enriched terms. **d** Volcano plot of TE subfamilies in cluster B regions, illustrating many more TE subfamilies become

upregulated than downregulated in TKO mMSCs (left). Barplot of subfamilies significantly upregulated in cluster B regions in TKO mMSC, indicating these upregulated TEs largely derive from the ERV1 and ERVK families of TEs (right). Upregulated subfamilies were selected based on adjusted p-value < 0.05, log2FoldChange > 1 and n > 3. * refers to *p*-value < 0.05 from a two-sided Wilcoxon rank-sum test. For the boxplots, boxes span the lower (first quartile) and upper quartiles (third quartile), median is indicated with a center line and whiskers extend to a maximum of 1.5 times the interquartile range. Source data are provided as a Source Data file.

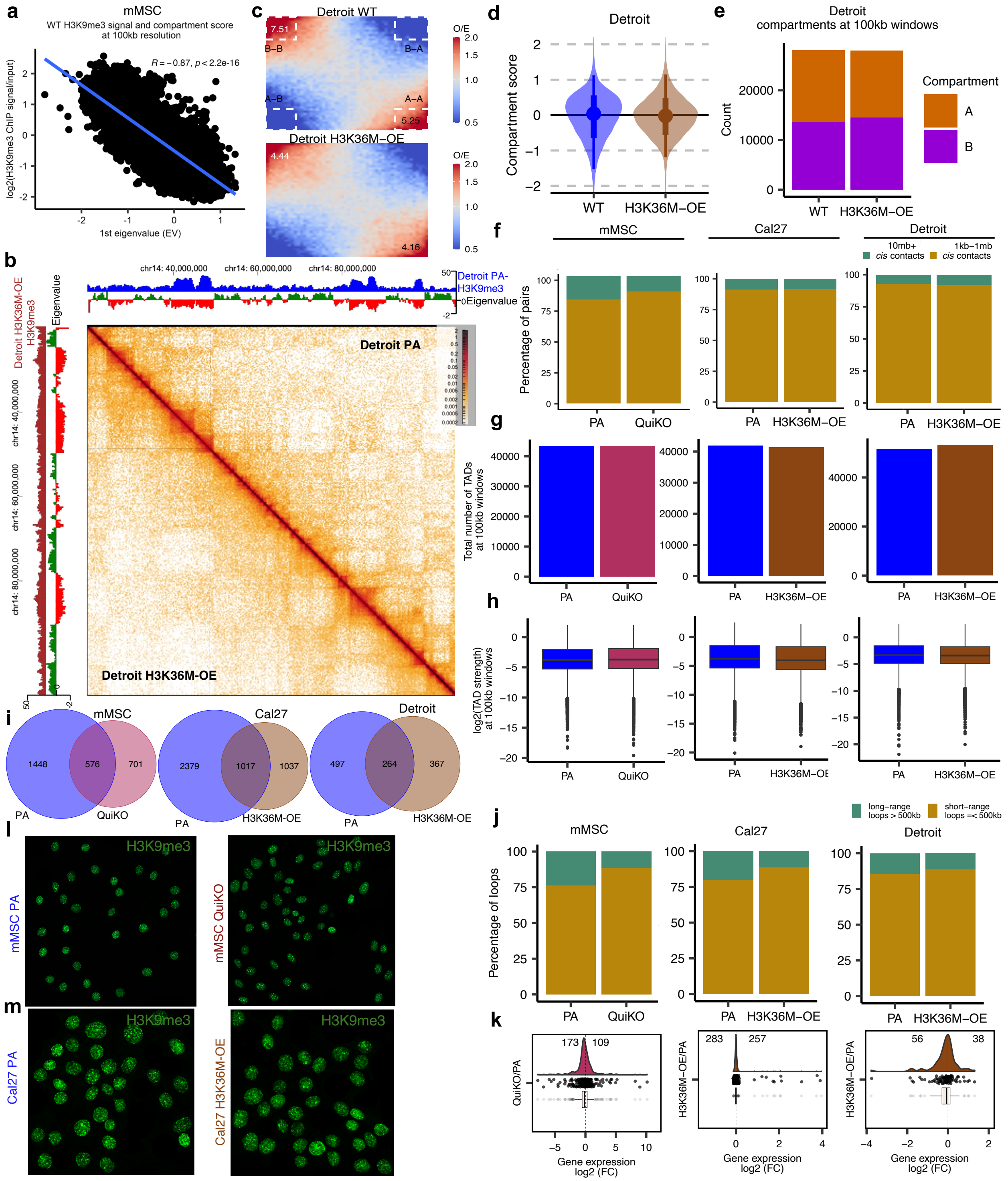

Supplementary Figure 8. Analysis of chromatin structure following H3K36me2 depletion. a Correlation plot depicting a strong negative linear correlation between H3K9me3 signal and the 1st eigenvalue. Pearson's correlation coefficient (*R*) and the associated *p*-value are reported. **b** Hi-C heatmaps illustrating reduced nuclear

compartmentalization in H3K36M-OE Detroit562. The blue (x-axis) and maroon (y-axis) tracks correspond to MS-normalized H3K9me3 for PA and H3K36M-OE respectively. **c** Saddleplots showing decreased A-A and B-B *cis* compartment interactions in H3K36M-OE Detroit562. **d** Violin-plots showing a shift of compartment scores towards zero, indicating decreased compartmentalization in H3K36M-OE Detroit562. n = 28020 compartments per condition. **e** Barplots illustrating a shift of A to B compartments in H3K36M-OE Detroit562. **f** Barplots of long-range *cis*-contacts, showing reduced long-range contacts (10 mb+) only in QuiKO mMSCs. **g** Barplots depicting the total number of TADs at 100kb windows, with no significant differences in QuiKO mMSCs or H3K36M-OE HNSCC compared to their respective parental controls. **h** Boxplots of TAD strength at 100kb windows, showing no differences between QuiKO mMSC and H3K36M-OE HNSCC compared to their respective parental controls. mMSC: n = 43350 TADs per condition; Cal27: n = 41973; Detroit562: n = 51667. **i** Venn diagrams of the number of loops at 25 kb resolution, depicting reduced number of loops in QuiKO mMSCs and H3K36M-OE HNSCC. **j** Barplots showing a greater proportion of long-range loops are diminished compared to short-range loops in QuiKO mMSCs and H3K36M-OE HNSCC. **k** Box- and violin-plots show target genes of enhancers located at the anchors of long-distance loops lost in H3K36me-deficient conditions are more frequently downregulated than upregulated (173 vs. 109 in mMSC, 283 vs. 257 in Cal27, and 56 vs. 38 in Detroit562) **l-m** Representative immunofluorescence images depicting loss of H3K9me3 foci at the periphery, with the remaining foci clustered towards the center of nuclei in QuiKO mMSCs and Cal27 H3K36M-OE. Nuclei were stained with anti-H3K9me3 antibody. For the boxplots, boxes span the first and third quartile, median is indicated with a center line and whiskers extend to 1.5 times the interquartile range. Source data are provided as a Source Data file.

Supplementary Table 1. SUV39H1 shRNA target sequences and manufacturer identifiers

shRNA identifier	Target sequence	Region
TRCN0000097439	GCCTTTGTACTCAGGAAAGAA	3'-UTR
TRCN0000097440	CGAGTCCGTATTGAATGCAAA	CDS
TRCN0000097441	CCTGCACAAGTTTGCCTACAA	CDS
TRCN0000097442	GTGCTGTAAATCTTCTTGGAA	CDS
TRCN0000097443	CTCTGCATCTTCCGCACTAAT	CDS

Supplementary Table 2. Antibodies

Antibody	Dilution	Source	Identifier/Catalogue no.
Rabbit monoclonal anti-H3K36me2	1:50	Cell Signaling Technology	C75H12, Cat # 2901
Rabbit polyclonal anti-H3K27ac	1:80	Diagenode	RRID:AB_2637079, Cat # C15410196
Rabbit monoclonal anti-H3K4me1	1:50	Cell Signaling Technology	D1A9, Cat # 5326S
Rabbit polyclonal anti-H3K4me3	1:90	Diagenode	Cat # C15410003-50
Mouse monoclonal anti-H3K27me1	1:100	Active Motif	RRID:AB_2715573, Cat # 61016
Rabbit monoclonal anti-H3K27me2	1:50	Cell Signaling Technology	D18C8, Cat # 9728
Rabbit monoclonal anti-H3K27me3	1:50	Cell Signaling Technology	RRID:AB_2616029, Cat # 9733
Rabbit polyclonal anti-H3K9me3	1:50	Abcam	Cat # ab8898
Rabbit monoclonal anti-SUV39H1	1:750	Cell Signaling Technology	Cat # 8729T
Mouse monoclonal anti-FLAG M2	1:5000	Sigma-Aldrich	Cat # F3165
Rabbit polyclonal anti-HP1	1:25	Cell Signaling Technology	Cat # 2616

Supplementary Table 3. Processed mass spectrometry data

Sample	H3K36me2	H3K36me3	H3K27me1	H3K27me2	H3K27me3	H3K4me1	H3K9me3
mMSC_PA_rep1	26.71068121	6.985385804	23.8970882	42.3271433	10.5210176	22.3261	5.0943
mMSC_PA_rep2	29.333102	8.315880447	28.3569629	37.1772451	7.828945522	25.5733	8.2274
mMSC_PA_rep3	26.70025097	6.738546053	24.2304288	42.6069494	10.17663415	22.5899	5.0602
mMSC_SETD2KO_rep1	39.67866997	1.061087822	23.2362329	57.1124903	7.71850479	21.9652	10.2455
mMSC_SETD2KO_rep2	37.84769395	1.055283327	21.4401442	59.4021509	10.32210569	29.8828	11.6881
mMSC_SETD2KO_rep3	37.84372192	1.020187262	22.7838136	49.2117573	7.943982672	28.7846	7.891
mMSC_NSD12_DKO_rep1	8.996929647	3.981035164	17.8823497	52.6571211	13.61900303	26.8013	5.6861
mMSC_NSD12_DKO_rep2	9.247317233	4.300172884	17.9403604	51.8838152	14.0462601	27.0217	6.2736
mMSC_H3K36M_OE_rep1	3.161928206	1.048270681	13.309736	56.7323101	15.02060477	21.4228	15.1897
mMSC_H3K36M_OE_rep2	2.634411737	1.047008778	14.4793288	55.106241	15.377008	29.4288	7.6538
mMSC_NSD12_SETD2_TKO_rep1	4.532196123	0.384516032	11.4800787	59.2969926	19.06762654	33.9875	5.4476
mMSC_NSD12_SETD2_TKO_rep2	4.727092463	0.341372293	11.8191298	58.2556046	16.44890625	29.0252	6.0284
mMSC_NSD12_SETD2_TKO_rep3	4.525062888	0.360940361	9.82875001	60.690542	20.09972406	26.756	10.9514
mMSC_NSD123_SETD2_QKO_rep1	0.023049694	0	8.99476839	63.1064066	21.48440155	32.7539	5.4823
mMSC_NSD123_SETD2_QKO_rep2	0.03624169	0.022024507	9.92685753	61.5060315	20.42434786	40.5426	6.082
mMSC_NSD123_SETD2_QKO_rep3	0.016754766	0	7.74647887	60.3844001	24.03694972	42.0321	5.7595
mMSC_NSD123_SETD2_ASH1L_QuiKO_rep1	2.53E-04	0	6.28361808	57.8559661	30.62498151	47.1286	4.9424
mMSC_NSD123_SETD2_ASH1L_QuiKO_rep2	0.027153183	0	8.09060343	59.3121752	25.3710612	42.182	4.4679
mMSC_NSD123_SETD2_ASH1L_QuiKO_rep3	8.81E-05	0	6.39676352	60.9461223	26.49484604	36.2042	4.7888
mMSC_PA_SUV39H1_WT_rep1	28.52471548	6.445196346	19.3226922	48.0452547	12.494311	11.2625	15.4494
mMSC_PA_SUV39H1_WT_rep2	28.12137138	6.652941648	19.6539356	46.5236583	11.730975	11.7436	14.9066
mMSC_PA_SUV39H1_KD_rep1	25.40324758	6.867601038	19.0228105	46.8832308	12.264872	12.3227	9.7437
mMSC_PA_SUV39H1_KD_rep2	25.32612785	6.869844104	17.3830689	47.8855503	12.827751	12.3695	11.0391
mMSC_PA_SUV39H1_OE_rep1	24.16651642	6.755797806	18.2150232	49.9526164	11.452184	12.0606	17.0197
mMSC_PA_SUV39H1_OE_rep2	24.92665918	6.807862466	18.5200866	49.5850444	11.453784	11.503	17.6753
mMSC_TKO_SUV39H1_KD_rep1	4.995422855	1.523876087	9.09407343	60.9135722	19.460618	19.4255	15.4795
mMSC_TKO_SUV39H1_KD_rep2	5.732152453	1.620291052	8.94845928	61.1146489	19.655762	20.6631	14.6127
mMSC_TKO_SUV39H1_OE_rep1	3.512358444	1.187424071	12.5571153	58.9031423	14.495632	14.7888	22.5934
mMSC_TKO_SUV39H1_OE_rep2	4.777914484	1.257254202	11.9929519	57.9911641	14.423662	14.636	24.4074
Cal27_PA	45.032885	2.7512989	16.842846	56.89816	12.89214	15.5083	20.5443
Cal27_H3K36M_OE	13.78447	0.4834152	8.624922	64.41646	17.73839	56.9904	12.3201
Detroit562_PA	40.600794	4.5145795	15.146947	57.87236	12.7435	17.589	25.5039
Detroit562_H3K36M_OE	7.729366	0.3650543	9.517769	61.79305	15.76538	22.0157	18.3274
