# Supplementary material for: H3K36 Methylation as a Guardian of Epigenome Integrity
Source: Nat Commun. 2025 Dec 11;16:11371. doi: 10.1038/s41467-025-66365-9 (PMC12727757; doi:10.1038/s41467-025-66365-9)
Supplement: Supplementary file 3 — Reporting Summary [file 41467_2025_66365_MOESM3_ESM.pdf]

nature portfolio

Corresponding author(s): Dr. Jacek Majewski

Last updated by author(s): Oct 23, 2025

Reporting Summary

Nature Portfolio wishes to improve the reproducibility of the work that we publish. This form provides structure for consistency and transparency in reporting. For further information on Nature Portfolio policies, see our Editorial Policies and the Editorial Policy Checklist.

Statistics

For all statistical analyses, confirm that the following items are present in the figure legend, table legend, main text, or Methods section.

n/a	Confirmed	
☐	☒	The exact sample size (*n*) for each experimental group/condition, given as a discrete number and unit of measurement
☐	☒	A statement on whether measurements were taken from distinct samples or whether the same sample was measured repeatedly
☐	☒	The statistical test(s) used AND whether they are one- or two-sided<br>*Only common tests should be described solely by name; describe more complex techniques in the Methods section.*
☒	☐	A description of all covariates tested
☐	☒	A description of any assumptions or corrections, such as tests of normality and adjustment for multiple comparisons
☐	☒	A full description of the statistical parameters including central tendency (e.g. means) or other basic estimates (e.g. regression coefficient) AND variation (e.g. standard deviation) or associated estimates of uncertainty (e.g. confidence intervals)
☐	☒	For null hypothesis testing, the test statistic (e.g. *F*, *t*, *r*) with confidence intervals, effect sizes, degrees of freedom and *P* value noted<br>*Give P values as exact values whenever suitable.*
☒	☐	For Bayesian analysis, information on the choice of priors and Markov chain Monte Carlo settings
☒	☐	For hierarchical and complex designs, identification of the appropriate level for tests and full reporting of outcomes
☐	☒	Estimates of effect sizes (e.g. Cohen's *d*, Pearson's *r*), indicating how they were calculated

Our web collection on statistics for biologists contains articles on many of the points above.

Software and code

Policy information about availability of computer code

Data collection	All software used for data collection is commercially available and stated in the Methods section. For RNA-seq, ATAC-seq, ChIP-seq, CUT&RUN, WGBS and Hi-C data collection, Illumina Hiseq 4000, NovaSeq 6000 or Illumina HiSeqX platform were used.<br>For collection of immunofluorescent images, sample coverslips were inverted onto permafluor mountant (epredia) on microscope slides, viewed and imaged on confocal LSM800. Z-stacks of 10 slices were taken and image projection (average setting) was built using Fiji (v.2.15.1). All images were taken under the same conditions, but laser strength and exposure were slightly adjusted for each image so that there is no saturation and ensuring similar fluorescence intensity in each image. This allowed a proper comparison of the spatial distribution of the foci. Images were filtered using ImageJ (v.1.54) for any nuclei that were dividing and any nuclei that were cutoff at the border of the images.
Data analysis	Visualization: ggplot2 (v.3.3.0), pyGenomeTracks (v.3.2.1), Trackplot (v.1.5.1), deepTools (v.3.3.1)<br>ChIP-Seq/CUT&RUN: BWA (v.0.7.17), Samtools (v.1.22.0), Samclip (v.0.2), Fastp (v.0.23.3), Bowtie (v.2.5.1), ChIPbinner (v.0.99.1), BEDTools (v.2.31.1), MACS2 (v.2.2.6), ROSE (v.1.0.0), Profileplyr (v.1.6.0)<br>ATAC-Seq: Trimmomatic (v.0.39), IDR (v.2.3.0)<br>RNA-Seq: STAR (v.2.5.3a), featureCounts (v.1.5.3), DESeq2 (v.1.26.0), apeglm (v.1.8.0), gplots (v.3.1.3), clusterProfiler (v.4.17.0)<br>WGBS: Bismark (v.0.24.2)<br>Hi-C: Pairtools (v.1.1.0), Cooler (v.0.10.0), Cooltools (v.0.4.0)<br>Immunofluorescent analysis: CellProfiler (v.4.2.6)<br>Details can be found in the Methods section under "Visualization", "ChIP-seq, CUT&RUN, ATAC-seq and RNA-seq processing and analysis", "WGBS processing and analysis", "Hi-C processing and analysis" and "Immunofluorescent staining and analysis".

For manuscripts utilizing custom algorithms or software that are central to the research but not yet described in published literature, software must be made available to editors and reviewers. We strongly encourage code deposition in a community repository (e.g. GitHub). See the Nature Portfolio guidelines for submitting code & software for further information.

Data

Policy information about availability of data

All manuscripts must include a data availability statement. This statement should provide the following information, where applicable:

- Accession codes, unique identifiers, or web links for publicly available datasets
- A description of any restrictions on data availability
- For clinical datasets or third party data, please ensure that the statement adheres to our policy

Previously published and publicly available ChIP-seq data can be accessed via the National Center for Biotechnology Information Gene Expression Omnibus (NCBI-GEO): H3K9me3 (parental) under accession number GSE118785 [https://www.ncbi.nlm.nih.gov/geo/query/acc.cgi?acc=GSE118785], H3K27me3 (parental, SETD2-KO, H3K36M, and NSD1-2-DKO samples) under accession number GSE160266 [https://www.ncbi.nlm.nih.gov/geo/query/acc.cgi?acc=GSE160266], and H3K36me2 (parental, ASH1L-KO, NSD3-KO, NSD1-2-SETD2-TKO, NSD1-2-3-SETD2-QKO, and NSD1-2-3-SETD2-ASH1L-QuiKO samples) under accession number GSE243566 [https://www.ncbi.nlm.nih.gov/geo/query/acc.cgi?acc=GSE243566]. Newly generated ChIP-seq, ATAC-seq, CUT&RUN, WGBS, RNA-seq and Hi-C data can be accessed under accession number GSE274367 [https://www.ncbi.nlm.nih.gov/geo/query/acc.cgi?acc=GSE274367]. Large H3K36me2 enhancer peaks identified in the mMSC NSD1/2-SETD2-TKO samples are available in the GitHub repository: (https://github.com/padilr1/H3K36me_guardian_epigenome_integrity_Padilla.git), located in the 'data' folder.

Research involving human participants, their data, or biological material

Policy information about studies with human participants or human data. See also policy information about sex, gender (identity/presentation), and sexual orientation and race, ethnicity and racism.

Reporting on sex and gender	Biological sex was not considered in the study design, as all experiments were performed using in vitro cultured mouse and human cell lines that do not exhibit sex-specific epigenetic differences relevant to the mechanistic focus of this study. The molecular pathways investigated—centered on H3K36 methylation and chromatin regulation—are not known to be sex-dependent in these systems.
Reporting on race, ethnicity, or other socially relevant groupings	*Please specify the socially constructed or socially relevant categorization variable(s) used in your manuscript and explain why they were used. Please note that such variables should not be used as proxies for other socially constructed/relevant variables (for example, race or ethnicity should not be used as a proxy for socioeconomic status).* *Provide clear definitions of the relevant terms used, how they were provided (by the participants/respondents, the researchers, or third parties), and the method(s) used to classify people into the different categories (e.g. self-report, census or administrative data, social media data, etc.)* *Please provide details about how you controlled for confounding variables in your analyses.*
Population characteristics	*Describe the covariate-relevant population characteristics of the human research participants (e.g. age, genotypic information, past and current diagnosis and treatment categories). If you filled out the behavioural & social sciences study design questions and have nothing to add here, write "See above."*
Recruitment	*Describe how participants were recruited. Outline any potential self-selection bias or other biases that may be present and how these are likely to impact results.*
Ethics oversight	*Identify the organization(s) that approved the study protocol.*

Note that full information on the approval of the study protocol must also be provided in the manuscript.

Field-specific reporting

Please select the one below that is the best fit for your research. If you are not sure, read the appropriate sections before making your selection.

☒ Life sciences ☐ Behavioural & social sciences ☐ Ecological, evolutionary & environmental sciences

For a reference copy of the document with all sections, see nature.com/documents/nr-reporting-summary-flat.pdf

Life sciences study design

All studies must disclose on these points even when the disclosure is negative.

Sample size	No statistical method was used to pre-determine sample sizes. Biological replicates (minimum of two per condition) were included for all statistical comparisons and high-throughput assays (ChIP-seq, CUT&RUN, ATAC-seq, RNA-seq and Hi-C), consistent with standard practices in genomics research and sufficient to ensure reproducibility of observed trend. Cross-species validation, using mMSC and HNSCC models, was performed to further support the observed outcomes. For immunofluorescence analysis, a minimum of 100 nuclei per condition was examined, and findings were reproduced in both mMSC, Cal27, and Detroit562 models.
Data exclusions	No data were excluded.
Replication	Key findings were consistently reproduced across multiple knockout conditions in mMSCs (DKO, TKO, QKO, and QuiKO). All major experiments (including ChIP-seq, CUT&RUN, ATAC-seq, RNA-seq and Hi-C) were independently performed at least twice with reproducible results.

	Furthermore, cross-species validation using both mMSC and HNSCC models reinforced the robustness and generality of the findings.
Randomization	Samples were allocated to groups according to genotype. No randomization was required. The sequencing of nucleic acid libraries are not affected by sample randomization.
Blinding	The experiments were not performed in a blinded manner, although the results were validated in biological replicates.

Reporting for specific materials, systems and methods

We require information from authors about some types of materials, experimental systems and methods used in many studies. Here, indicate whether each material, system or method listed is relevant to your study. If you are not sure if a list item applies to your research, read the appropriate section before selecting a response.

Materials & experimental systems

n/a	Involved in the study
☐	☒ Antibodies
☐	☒ Eukaryotic cell lines
☒	☐ Palaeontology and archaeology
☒	☐ Animals and other organisms
☒	☐ Clinical data
☒	☐ Dual use research of concern
☒	☐ Plants

Methods

n/a	Involved in the study
☐	☒ ChIP-seq
☒	☐ Flow cytometry
☒	☐ MRI-based neuroimaging

Antibodies

Antibodies used	For Western blots, the following primary antibodies were used: anti-FLAG M2 (Sigma-Aldrich, F3165), anti-SUV39H1 (CST, 8729T)<br><br>For ChIP-seq and CUT&RUN the following primary antibodies were used: anti-H3K36me2 (CST, 2901)(1:50), anti-H3K27ac (Diagenode, C15410196)(1:80), anti-H3K4me1 (CST, D1A9, 5326S)(1:50), anti-H3K4me3 (Diagenode, C15410003-50)(1:90), anti-H3K27me1 (Active Motif, 61016)(1:100), anti-H3K27me2 (CST, 9728)(1:50), anti-H3K27me3 (CST, 9733)(1:50), anti-H3K9me3 (Abcam, ab8898)(1:57), anti-FLAG M2 (Sigma-Aldrich, F3165), anti-HP1 (CST, 2616)(1:25)
Validation	For ChIP, the specificity of antibodies was tested by dot blot on a histone modification peptide array. Antibodies used have been used in the literature and also validated by manufacturers.

Eukaryotic cell lines

Policy information about cell lines and Sex and Gender in Research

Cell line source(s)	C3H10T1/2 (ATCC, CCL-226)<br>Cal27 (ATCC, CRL-2095)<br>Detroit562 (ATCC, CCL-138)
Authentication	Cell lines have been authenticated as unique by distinct DNA methylation, RNA-seq, and chromatin patterns. The identity of cell lines were checked by microsatellite typing (DNA fingerprinting) and variant calling. CRISPR/Cas9 edited clones were previously verified by targeted resequencing by MiSeq to confirm specific genome editing events (as described in Shipman et al. Genome Biology 2024). No new CRISPR edited cell lines were established in this study.
Mycoplasma contamination	All cell lines tested negative for mycoplasma contamination.
Commonly misidentified lines<br>(See ICLAC register)	No commonly misidentified lines were used in this study.

Plants

Seed stocks	*Report on the source of all seed stocks or other plant material used. If applicable, state the seed stock centre and catalogue number. If plant specimens were collected from the field, describe the collection location, date and sampling procedures.*
Novel plant genotypes	*Describe the methods by which all novel plant genotypes were produced. This includes those generated by transgenic approaches, gene editing, chemical/radiation-based mutagenesis and hybridization. For transgenic lines, describe the transformation method, the number of independent lines analyzed and the generation upon which experiments were performed. For gene-edited lines, describe the editor used, the endogenous sequence targeted for editing, the targeting guide RNA sequence (if applicable) and how the editor was applied.*
Authentication	*Describe any authentication procedures for each seed stock used or novel genotype generated. Describe any experiments used to assess the effect of a mutation and, where applicable, how potential secondary effects (e.g. second site T-DNA insertions, mosiacism, off-target gene editing) were examined.*

ChIP-seq

Data deposition

☒ Confirm that both raw and final processed data have been deposited in a public database such as GEO.

☒ Confirm that you have deposited or provided access to graph files (e.g. BED files) for the called peaks.

Data access links
May remain private before publication.

ChIP-seq data can be accessed under accession number GSE274367
ChIP-seq peak file for C3H10T1-2 NSD12-SETD2-TKO H3K36me2 can be found in the 'data' folder at: https://github.com/padilr1/H3K36me_guardian_epigenome_integrity_Padilla.git

Files in database submission

C3H10T1-2_H3K36M_ChIPseq_H3K27me1_batch1.cpm.bw
C3H10T1-2_H3K36M_ChIPseq_H3K27me2_batch1.cpm.bw
C3H10T1-2_NSD1-2-3-SETD2-ASH1L-QuiKO_ChIPseq_H3K27ac_rep1_batch2.cpm.bw
C3H10T1-2_NSD1-2-3-SETD2-ASH1L-QuiKO_ChIPseq_H3K27ac_rep2_batch2.cpm.bw
C3H10T1-2_NSD1-2-3-SETD2-ASH1L-QuiKO_ChIPseq_H3K27ac_rep3_batch2.cpm.bw
C3H10T1-2_NSD1-2-3-SETD2-ASH1L-QuiKO_ChIPseq_H3K4me3_rep1_batch2.cpm.bw
C3H10T1-2_NSD1-2-3-SETD2-ASH1L-QuiKO_ChIPseq_H3K4me3_rep2_batch2.cpm.bw
C3H10T1-2_NSD1-2-3-SETD2-ASH1L-QuiKO_ChIPseq_H3K4me3_rep3_batch2.cpm.bw
C3H10T1-2_NSD1-2-3-SETD2-QKO_ChIPseq_H3K27ac_rep1_batch2.cpm.bw
C3H10T1-2_NSD1-2-3-SETD2-QKO_ChIPseq_H3K27ac_rep2_batch2.cpm.bw
C3H10T1-2_NSD1-2-3-SETD2-QKO_ChIPseq_H3K27ac_rep3_batch2.cpm.bw
C3H10T1-2_NSD1-2-3-SETD2-QKO_ChIPseq_H3K4me3_rep1_batch2.cpm.bw
C3H10T1-2_NSD1-2-3-SETD2-QKO_ChIPseq_H3K4me3_rep2_batch2.cpm.bw
C3H10T1-2_NSD1-2-3-SETD2-QKO_ChIPseq_H3K4me3_rep3_batch2.cpm.bw
C3H10T1-2_NSD1-2-DKO_ChIPseq_H3K27me1_batch1.cpm.bw
C3H10T1-2_NSD1-2-DKO_ChIPseq_H3K27me2_batch1.cpm.bw
C3H10T1-2_NSD1-2-DKO_ChIPseq_H3K9me3_batch1.cpm.bw
C3H10T1-2_NSD1-2-SETD2-TKO_ChIPseq_H3K27ac_rep1_batch2.cpm.bw
C3H10T1-2_NSD1-2-SETD2-TKO_ChIPseq_H3K27ac_rep2_batch2.cpm.bw
C3H10T1-2_NSD1-2-SETD2-TKO_ChIPseq_H3K27ac_rep3_batch2.cpm.bw
C3H10T1-2_NSD1-2-SETD2-TKO_ChIPseq_H3K27me1_batch1.cpm.bw
C3H10T1-2_NSD1-2-SETD2-TKO_ChIPseq_H3K27me2_batch1.cpm.bw
C3H10T1-2_NSD1-2-SETD2-TKO_ChIPseq_H3K27me3_batch1.cpm.bw
C3H10T1-2_NSD1-2-SETD2-TKO_ChIPseq_H3K4me3_rep1_batch2.cpm.bw
C3H10T1-2_NSD1-2-SETD2-TKO_ChIPseq_H3K4me3_rep2_batch2.cpm.bw
C3H10T1-2_NSD1-2-SETD2-TKO_ChIPseq_H3K4me3_rep3_batch2.cpm.bw
C3H10T1-2_NSD1-2-SETD2-TKO_ChIPseq_H3K9me3_batch1.cpm.bw
C3H10T1-2_Parental_ChIPseq_H3K27ac_rep1_batch2.cpm.bw
C3H10T1-2_Parental_ChIPseq_H3K27ac_rep2_batch2.cpm.bw
C3H10T1-2_Parental_ChIPseq_H3K27ac_rep3_batch2.cpm.bw
C3H10T1-2_Parental_ChIPseq_H3K27me1_batch1.cpm.bw
C3H10T1-2_Parental_ChIPseq_H3K27me2_batch1.cpm.bw
C3H10T1-2_Parental_ChIPseq_H3K4me3_rep1_batch2.cpm.bw
C3H10T1-2_Parental_ChIPseq_H3K4me3_rep2_batch2.cpm.bw
C3H10T1-2_Parental_ChIPseq_H3K4me3_rep3_batch2.cpm.bw
C3H10T1-2_SETD2KO_ChIPseq_H3K27me1_batch1.cpm.bw
C3H10T1-2_SETD2KO_ChIPseq_H3K27me2_batch1.cpm.bw
HNSCC_Cal27_H3K36M_ChIPseq_H3K9me3.cpm.bw
HNSCC_Cal27_Parental_sgCtrl_ChIPseq_H3K9me3.cpm.bw
C3H10T1-2_PA_SUV39H1_FLAG.cpm.bw
C3H10T1-2_TKO_SUV39H1_FLAG.cpm.bw
C3H10T1-2_PA_HP1.cpm.bw
C3H10T1-2_TKO_HP1.cpm.bw
HNSCC_Cal27_Parental_ChIPseq_H3K27me3_batch2.cpm.bw
HNSCC_Cal27_H3K36M-OE_ChIPseq_H3K27me3_batch2.cpm.bw
HNSCC_Cal27_Parental_ChIPseq_H3K27ac_rep1_batch2.cpm.bw
HNSCC_Cal27_Parental_ChIPseq_H3K27ac_rep2_batch2.cpm.bw
HNSCC_Detroit562_H3K36M_OE_ChIPseq_H3K9me3.cpm.bw
HNSCC_Cal27_Parental_sgCtrl_ChIPseq_H3K9me3.cpm.bw
HNSCC_Detroit562_Parental_sgCtrl_ChIPseq_H3K27me3.cpm.bw
HNSCC_Detroit562_H3K36M_OE_ChIPseq_H3K27me3.cpm.bw
HNSCC_Detroit562_Parental_sgCtrl_ChIPseq_H3K27ac.cpm.bw
HNSCC_Detroit562_Parental_ChIPseq_H3K27ac.cpm.bw
HNSCC_Cal27_H3K36M_OE_ChIPseq_H3K27ac_rep1_batch2.cpm.bw
HNSCC_Cal27_H3K36M_OE_ChIPseq_H3K27ac_rep2_batch2.cpm.bw
HNSCC_Detroit562_H3K36M_OE_ChIPseq_H3K27ac_rep1.cpm.bw
HNSCC_Detroit562_H3K36M_OE_ChIPseq_H3K27ac_rep2.cpm.bw
C3H10T1-2_H3K36M_ChIPseq_H3K27me1_batch1.fastq.gz
C3H10T1-2_H3K36M_ChIPseq_H3K27me2_batch1.fastq.gz
C3H10T1-2_H3K36M_ChIPseq_input_batch1.fastq.gz
C3H10T1-2_NSD1-2-3-SETD2-ASH1L-QuiKO_ChIPseq_H3K27ac_rep1_batch2.R1.fq.gz
C3H10T1-2_NSD1-2-3-SETD2-ASH1L-QuiKO_ChIPseq_H3K27ac_rep2_batch2.R1.fq.gz

C3H10T1-2_NSD1-2-3-SETD2-ASH1L-QuiKO_ChIPseq_H3K27ac_rep3_batch2.R1.fq.gz
C3H10T1-2_NSD1-2-3-SETD2-ASH1L-QuiKO_ChIPseq_H3K4me3_rep1_batch2.R1.fq.gz
C3H10T1-2_NSD1-2-3-SETD2-ASH1L-QuiKO_ChIPseq_H3K4me3_rep2_batch2.R1.fq.gz
C3H10T1-2_NSD1-2-3-SETD2-ASH1L-QuiKO_ChIPseq_H3K4me3_rep3_batch2.R1.fq.gz
C3H10T1-2_NSD1-2-3-SETD2-ASH1L-QuiKO_ChIPseq_input_rep1_batch2.R1.fq.gz
C3H10T1-2_NSD1-2-3-SETD2-ASH1L-QuiKO_ChIPseq_input_rep2_batch2.R1.fq.gz
C3H10T1-2_NSD1-2-3-SETD2-ASH1L-QuiKO_ChIPseq_input_rep3_batch2.R1.fq.gz
C3H10T1-2_NSD1-2-3-SETD2-QKO_ChIPseq_H3K27ac_rep1_batch2.R1.fq.gz
C3H10T1-2_NSD1-2-3-SETD2-QKO_ChIPseq_H3K27ac_rep2_batch2.R1.fq.gz
C3H10T1-2_NSD1-2-3-SETD2-QKO_ChIPseq_H3K27ac_rep3_batch2.R1.fq.gz
C3H10T1-2_NSD1-2-3-SETD2-QKO_ChIPseq_H3K4me3_rep1_batch2.R1.fq.gz
C3H10T1-2_NSD1-2-3-SETD2-QKO_ChIPseq_H3K4me3_rep2_batch2.R1.fq.gz
C3H10T1-2_NSD1-2-3-SETD2-QKO_ChIPseq_H3K4me3_rep3_batch2.R1.fq.gz
C3H10T1-2_NSD1-2-3-SETD2-QKO_ChIPseq_input_rep1_batch2.R1.fq.gz
C3H10T1-2_NSD1-2-3-SETD2-QKO_ChIPseq_input_rep2_batch2.R1.fq.gz
C3H10T1-2_NSD1-2-3-SETD2-QKO_ChIPseq_input_rep3_batch2.R1.fq.gz
C3H10T1-2_NSD1-2-DKO_ChIPseq_H3K27me1_batch1.fastq.gz
C3H10T1-2_NSD1-2-DKO_ChIPseq_H3K27me2_batch1.fastq.gz
C3H10T1-2_NSD1-2-DKO_ChIPseq_H3K9me3_batch1.fastq.gz
C3H10T1-2_NSD1-2-DKO_ChIPseq_input_batch1.fastq.gz
C3H10T1-2_NSD1-2-SETD2-TKO_ChIPseq_H3K27ac_rep1_batch2.R1.fq.gz
C3H10T1-2_NSD1-2-SETD2-TKO_ChIPseq_H3K27ac_rep2_batch2.R1.fq.gz
C3H10T1-2_NSD1-2-SETD2-TKO_ChIPseq_H3K27ac_rep3_batch2.R1.fq.gz
C3H10T1-2_NSD1-2-SETD2-TKO_ChIPseq_H3K27me1_batch1.fastq.gz
C3H10T1-2_NSD1-2-SETD2-TKO_ChIPseq_H3K27me2_batch1.fastq.gz
C3H10T1-2_NSD1-2-SETD2-TKO_ChIPseq_H3K27me3_batch1.fastq.gz
C3H10T1-2_NSD1-2-SETD2-TKO_ChIPseq_H3K4me3_rep1_batch2.R1.fq.gz
C3H10T1-2_NSD1-2-SETD2-TKO_ChIPseq_H3K4me3_rep2_batch2.R1.fq.gz
C3H10T1-2_NSD1-2-SETD2-TKO_ChIPseq_H3K4me3_rep3_batch2.R1.fq.gz
C3H10T1-2_NSD1-2-SETD2-TKO_ChIPseq_H3K9me3_batch1.fastq.gz
C3H10T1-2_NSD1-2-SETD2-TKO_ChIPseq_input_batch1.fastq.gz
C3H10T1-2_NSD1-2-SETD2-TKO_ChIPseq_input_rep1-2_batch2.R1.fq.gz
C3H10T1-2_NSD1-2-SETD2-TKO_ChIPseq_input_rep3_batch2.R1.fq.gz
C3H10T1-2_Parental_ChIPseq_H3K27ac_rep1_batch2.R1.fq.gz
C3H10T1-2_Parental_ChIPseq_H3K27ac_rep2_batch2.R1.fq.gz
C3H10T1-2_Parental_ChIPseq_H3K27ac_rep3_batch2.R1.fq.gz
C3H10T1-2_Parental_ChIPseq_H3K27me1_batch1.fastq.gz
C3H10T1-2_Parental_ChIPseq_H3K27me2_batch1.fastq.gz
C3H10T1-2_Parental_ChIPseq_H3K4me3_rep1_batch2.R1.fq.gz
C3H10T1-2_Parental_ChIPseq_H3K4me3_rep2_batch2.R1.fq.gz
C3H10T1-2_Parental_ChIPseq_H3K4me3_rep3_batch2.R1.fq.gz
C3H10T1-2_Parental_ChIPseq_input_batch1.fastq.gz
C3H10T1-2_Parental_ChIPseq_input_rep1-2_batch2.R1.fq.gz
C3H10T1-2_Parental_ChIPseq_input_rep3_batch2.R1.fq.gz
C3H10T1-2_SETD2KO_ChIPseq_H3K27me1_batch1.fastq.gz
C3H10T1-2_SETD2KO_ChIPseq_H3K27me2_batch1.fastq.gz
C3H10T1-2_SETD2KO_ChIPseq_input_batch1.fastq.gz
HNSCC_Cal27_H3K36M_ChIPseq_H3K9me3.R1.fq.gz
HNSCC_Cal27_H3K36M_ChIPseq_input.fq.gz
HNSCC_Cal27_Parental_sgCtrl_ChIPseq_H3K9me3.R1.fq.gz
HNSCC_Cal27_Parental_sgCtrl_ChIPseq_input.R1.fq.gz
C3H10T1-2_NSD1-2-3-SETD2-ASH1L-QuiKO_ChIPseq_H3K27ac_rep1_batch2.R2.fq.gz
C3H10T1-2_NSD1-2-3-SETD2-ASH1L-QuiKO_ChIPseq_H3K27ac_rep2_batch2.R2.fq.gz
C3H10T1-2_NSD1-2-3-SETD2-ASH1L-QuiKO_ChIPseq_H3K27ac_rep3_batch2.R2.fq.gz
C3H10T1-2_NSD1-2-3-SETD2-ASH1L-QuiKO_ChIPseq_H3K4me3_rep1_batch2.R2.fq.gz
C3H10T1-2_NSD1-2-3-SETD2-ASH1L-QuiKO_ChIPseq_H3K4me3_rep2_batch2.R2.fq.gz
C3H10T1-2_NSD1-2-3-SETD2-ASH1L-QuiKO_ChIPseq_H3K4me3_rep3_batch2.R2.fq.gz
C3H10T1-2_NSD1-2-3-SETD2-ASH1L-QuiKO_ChIPseq_input_rep1_batch2.R2.fq.gz
C3H10T1-2_NSD1-2-3-SETD2-ASH1L-QuiKO_ChIPseq_input_rep2_batch2.R2.fq.gz
C3H10T1-2_NSD1-2-3-SETD2-ASH1L-QuiKO_ChIPseq_input_rep3_batch2.R2.fq.gz
C3H10T1-2_NSD1-2-3-SETD2-QKO_ChIPseq_H3K27ac_rep1_batch2.R2.fq.gz
C3H10T1-2_NSD1-2-3-SETD2-QKO_ChIPseq_H3K27ac_rep2_batch2.R2.fq.gz
C3H10T1-2_NSD1-2-3-SETD2-QKO_ChIPseq_H3K27ac_rep3_batch2.R2.fq.gz
C3H10T1-2_NSD1-2-3-SETD2-QKO_ChIPseq_H3K4me3_rep1_batch2.R2.fq.gz
C3H10T1-2_NSD1-2-3-SETD2-QKO_ChIPseq_H3K4me3_rep2_batch2.R2.fq.gz
C3H10T1-2_NSD1-2-3-SETD2-QKO_ChIPseq_H3K4me3_rep3_batch2.R2.fq.gz
C3H10T1-2_NSD1-2-3-SETD2-QKO_ChIPseq_input_rep1_batch2.R2.fq.gz
C3H10T1-2_NSD1-2-3-SETD2-QKO_ChIPseq_input_rep2_batch2.R2.fq.gz
C3H10T1-2_NSD1-2-3-SETD2-QKO_ChIPseq_input_rep3_batch2.R2.fq.gz
C3H10T1-2_NSD1-2-SETD2-TKO_ChIPseq_H3K27ac_rep1_batch2.R2.fq.gz
C3H10T1-2_NSD1-2-SETD2-TKO_ChIPseq_H3K27ac_rep2_batch2.R2.fq.gz
C3H10T1-2_NSD1-2-SETD2-TKO_ChIPseq_H3K27ac_rep3_batch2.R2.fq.gz
C3H10T1-2_NSD1-2-SETD2-TKO_ChIPseq_H3K4me3_rep1_batch2.R2.fq.gz
C3H10T1-2_NSD1-2-SETD2-TKO_ChIPseq_H3K4me3_rep2_batch2.R2.fq.gz
C3H10T1-2_NSD1-2-SETD2-TKO_ChIPseq_H3K4me3_rep3_batch2.R2.fq.gz
C3H10T1-2_NSD1-2-SETD2-TKO_ChIPseq_input_rep1-2_batch2.R2.fq.gz

C3H10T1-2_NSD1-2-SETD2-TKO_ChIPseq_input_rep3_batch2.R2.fq.gz
C3H10T1-2_Parental_ChIPseq_H3K27ac_rep1_batch2.R2.fq.gz
C3H10T1-2_Parental_ChIPseq_H3K27ac_rep2_batch2.R2.fq.gz
C3H10T1-2_Parental_ChIPseq_H3K27ac_rep3_batch2.R2.fq.gz
C3H10T1-2_Parental_ChIPseq_H3K4me3_rep1_batch2.R2.fq.gz
C3H10T1-2_Parental_ChIPseq_H3K4me3_rep2_batch2.R2.fq.gz
C3H10T1-2_Parental_ChIPseq_H3K4me3_rep3_batch2.R2.fq.gz
C3H10T1-2_Parental_ChIPseq_input_rep1-2_batch2.R2.fq.gz
C3H10T1-2_Parental_ChIPseq_input_rep3_batch2.R2.fq.gz
HNSCC_Cal27_H3K36M_ChIPseq_H3K9me3.R2.fq.gz
HNSCC_Cal27_Parental_sgCtrl_ChIPseq_H3K9me3.R2.fq.gz
HNSCC_Cal27_Parental_sgCtrl_ChIPseq_input.R2.fq.gz
C3H10T1-2_PA_SUV39H1_FLAG.R1.fq.gz
C3H10T1-2_TKO_SUV39H1_FLAG.R1.fq.gz
C3H10T1-2_PA_SUV39H1_FLAG_input.R1.fq.gz
C3H10T1-2_TKO_SUV39H1_FLAG_input.R1.fq.gz
C3H10T1-2_PA_HP1.R1.fq.gz
C3H10T1-2_PA_HP1_input.R1.fq.gz
C3H10T1-2_TKO_HP1.R1.fq.gz
C3H10T1-2_TKO_HP1_input.R1.fq.gz
HNSCC_Cal27_Parental_ChIPseq_input_batch2.R1.fq.gz
HNSCC_Cal27_Parental_ChIPseq_H3K27me3_batch2.R1.fq.gz
HNSCC_Cal27_H3K36M-OE_ChIPseq_H3K27me3_batch2.R1.fq.gz
HNSCC_Cal27_Parental_ChIPseq_H3K27ac_rep1_batch2.R1.fq.gz
HNSCC_Cal27_Parental_ChIPseq_H3K27ac_rep2_batch2.R1.fq.gz
HNSCC_Cal27_H3K36M_OE_ChIPseq_H3K27ac_rep1_batch2.R1.fq.gz
HNSCC_Cal27_H3K36M_OE_input_batch2.R1.fq.gz
HNSCC_Detroit562_H3K36M_OE_ChIPseq_H3K9me3.R1.fq.gz
HNSCC_Detroit562_H3K36M_OE_ChIPseq_input.R1.fq.gz
HNSCC_Detroit562_Parental_sgCtrl_ChIPseq_H3K9me3.R1.fq.gz
HNSCC_Detroit562_Parental_sgCtrl_ChIPseq_input.R1.fq.gz
HNSCC_Detroit562_Parental_sgCtrl_ChIPseq_H3K27me3.R1.fq.gz
HNSCC_Detroit562_H3K36M_OE_ChIPseq_H3K27me3.R1.fq.gz
HNSCC_Detroit562_Parental_sgCtrl_ChIPseq_H3K27ac.R1.fq.gz
HNSCC_Detroit562_Parental_ChIPseq_H3K27ac.R1.fq.gz
HNSCC_Detroit562_H3K36M_OE_ChIPseq_H3K27ac_rep1.R1.fq.gz
C3H10T1-2_PA_SUV39H1_FLAG.R2.fq.gz
C3H10T1-2_TKO_SUV39H1_FLAG.R2.fq.gz
C3H10T1-2_PA_SUV39H1_FLAG_input.R2.fq.gz
C3H10T1-2_TKO_SUV39H1_FLAG_input.R2.fq.gz
C3H10T1-2_PA_HP1.R2.fq.gz
C3H10T1-2_PA_HP1_input.R2.fq.gz
C3H10T1-2_TKO_HP1.R2.fq.gz
C3H10T1-2_TKO_HP1_input.R2.fq.gz
HNSCC_Cal27_Parental_ChIPseq_input_batch2.R2.fq.gz
HNSCC_Cal27_Parental_ChIPseq_H3K27me3_batch2.R2.fq.gz
HNSCC_Cal27_H3K36M-OE_ChIPseq_H3K27me3_batch2.R2.fq.gz
HNSCC_Cal27_Parental_ChIPseq_H3K27ac_rep1_batch2.R2.fq.gz
HNSCC_Cal27_Parental_ChIPseq_H3K27ac_rep2_batch2.R2.fq.gz
HNSCC_Cal27_H3K36M_OE_ChIPseq_H3K27ac_rep1_batch2.R2.fq.gz
HNSCC_Cal27_H3K36M_OE_input_batch2.R2.fq.gz
HNSCC_Detroit562_H3K36M_OE_ChIPseq_H3K9me3.R2.fq.gz
HNSCC_Detroit562_H3K36M_OE_ChIPseq_input.R2.fq.gz
HNSCC_Detroit562_Parental_sgCtrl_ChIPseq_H3K9me3.R2.fq.gz
HNSCC_Detroit562_Parental_sgCtrl_ChIPseq_input.R2.fq.gz
HNSCC_Detroit562_Parental_sgCtrl_ChIPseq_H3K27me3.R2.fq.gz
HNSCC_Detroit562_H3K36M_OE_ChIPseq_H3K27me3.R2.fq.gz
HNSCC_Detroit562_Parental_sgCtrl_ChIPseq_H3K27ac.R2.fq.gz
HNSCC_Detroit562_Parental_ChIPseq_H3K27ac.R2.fq.gz
HNSCC_Detroit562_H3K36M_OE_ChIPseq_H3K27ac_rep1.R2.fq.gz
HNSCC_Cal27_H3K36M_OE_ChIPseq_H3K27ac_rep2_batch2.R1.fq.gz
HNSCC_Cal27_H3K36M_OE_ChIPseq_H3K27ac_rep2_batch2.R2.fq.gz
HNSCC_Detroit562_H3K36M_OE_ChIPseq_H3K27ac_rep2.R1.fq.gz
HNSCC_Detroit562_H3K36M_OE_ChIPseq_H3K27ac_rep2.R2.fq.gz
HNSCC_Detroit562_Parental_ChIPseq_input.R1.fq.gz
HNSCC_Detroit562_Parental_ChIPseq_input.R2.fq.gz

Genome browser session
(e.g. UCSC)

Not applicable - data visualization was performed using IGV.

Methodology

Replicates

For H3K27ac and H3K4me3 ChIP-seq profiling in C3H10T1/2 cells, three biological replicates were used per condition. Correlation matrix heatmaps of genome-wide signal confirmed strong reproducibility, with replicates clustering by condition. In Cal-27 and

Detroit562 cells, two biological replicates were used for each parental condition, while two technical replicates were generated for the H3K36M-OE condition. For all other ChIP-seq experiments, single replicates were used per condition due to the inclusion of multiple knockout conditions and the use of cross-species validation (C3H10T1/2 and HNSCC models) to support reproducibility.

Sequencing depth

ChIP-seq: 50bp, single-end, filtering was performed to remove low-quality and multi-mapping reads

C3H10T1-2_Parental_ChIPseq_input_batch1: 24386336
C3H10T1-2_SETD2KO_ChIPseq_input_batch1: 26261116
C3H10T1-2_H3K36M_ChIPseq_input_batch1: 7785847
C3H10T1-2_NSD1-2-DKO_ChIPseq_input_batch1: 33748936
C3H10T1-2_NSD1-2-SETD2-TKO_ChIPseq_input_batch1: 12166154
C3H10T1-2_Parental_ChIPseq_H3K27me1_batch1: 48178089
C3H10T1-2_SETD2KO_ChIPseq_H3K27me1_batch1: 48688105
C3H10T1-2_H3K36M_ChIPseq_H3K27me1_batch1: 39042163
C3H10T1-2_NSD1-2-DKO_ChIPseq_H3K27me1_batch1: 40181619
C3H10T1-2_NSD1-2-SETD2-TKO_ChIPseq_H3K27me1_batch1: 37841303
C3H10T1-2_Parental_ChIPseq_H3K27me2_batch1: 47089138
C3H10T1-2_SETD2KO_ChIPseq_H3K27me2_batch1: 32762903
C3H10T1-2_H3K36M_ChIPseq_H3K27me2_batch1: 36668318
C3H10T1-2_NSD1-2-DKO_ChIPseq_H3K27me2_batch1: 41863900
C3H10T1-2_NSD1-2-SETD2-TKO_ChIPseq_H3K27me2_batch1: 41430716
C3H10T1-2_NSD1-2-SETD2-TKO_ChIPseq_H3K27me3_batch1: 42477076
C3H10T1-2_NSD1-2-DKO_ChIPseq_H3K9me3_batch1: 24033001
C3H10T1-2_NSD1-2-SETD2-TKO_ChIPseq_H3K9me3_batch1: 24299097

ChIP-seq: 100bp, paired-end, filtering was performed to remove low-quality and multi-mapping reads

C3H10T1-2_Parental_ChIPseq_input_rep1-2_batch2: 28011758
C3H10T1-2_Parental_ChIPseq_input_rep3_batch2: 24044153
C3H10T1-2_NSD1-2-SETD2-TKO_ChIPseq_input_rep1-2_batch2: 21014119
C3H10T1-2_NSD1-2-SETD2-TKO_ChIPseq_input_rep3_batch2: 30989566
C3H10T1-2_NSD1-2-3-SETD2-QKO_ChIPseq_input_rep1_batch2: 28972623
C3H10T1-2_NSD1-2-3-SETD2-QKO_ChIPseq_input_rep2_batch2: 20812630
C3H10T1-2_NSD1-2-3-SETD2-QKO_ChIPseq_input_rep3_batch2: 29900133
C3H10T1-2_NSD1-2-3-SETD2-ASH1L-QuiKO_ChIPseq_input_rep1_batch2: 17784558
C3H10T1-2_NSD1-2-3-SETD2-ASH1L-QuiKO_ChIPseq_input_rep2_batch2: 30948788
C3H10T1-2_NSD1-2-3-SETD2-ASH1L-QuiKO_ChIPseq_input_rep3_batch2: 23565125
C3H10T1-2_Parental_ChIPseq_H3K27ac_rep1_batch2: 52700481
C3H10T1-2_Parental_ChIPseq_H3K27ac_rep2_batch2: 55140887
C3H10T1-2_Parental_ChIPseq_H3K27ac_rep3_batch2: 53750784
C3H10T1-2_NSD1-2-SETD2-TKO_ChIPseq_H3K27ac_rep1_batch2: 39527381
C3H10T1-2_NSD1-2-SETD2-TKO_ChIPseq_H3K27ac_rep2_batch2: 74022817
C3H10T1-2_NSD1-2-SETD2-TKO_ChIPseq_H3K27ac_rep3_batch2: 57514200
C3H10T1-2_NSD1-2-3-SETD2-QKO_ChIPseq_H3K27ac_rep1_batch2: 51007546
C3H10T1-2_NSD1-2-3-SETD2-QKO_ChIPseq_H3K27ac_rep2_batch2: 50027806
C3H10T1-2_NSD1-2-3-SETD2-QKO_ChIPseq_H3K27ac_rep3_batch2: 61975575
C3H10T1-2_NSD1-2-3-SETD2-ASH1L-QuiKO_ChIPseq_H3K27ac_rep1_batch2: 38949516
C3H10T1-2_NSD1-2-3-SETD2-ASH1L-QuiKO_ChIPseq_H3K27ac_rep2_batch2: 46009073
C3H10T1-2_NSD1-2-3-SETD2-ASH1L-QuiKO_ChIPseq_H3K27ac_rep3_batch2: 81902312
C3H10T1-2_Parental_ChIPseq_H3K4me3_rep1_batch2: 46409035
C3H10T1-2_Parental_ChIPseq_H3K4me3_rep2_batch2: 64261109
C3H10T1-2_Parental_ChIPseq_H3K4me3_rep3_batch2: 41447045
C3H10T1-2_NSD1-2-SETD2-TKO_ChIPseq_H3K4me3_rep1_batch2: 32566941
C3H10T1-2_NSD1-2-SETD2-TKO_ChIPseq_H3K4me3_rep2_batch2: 63342469
C3H10T1-2_NSD1-2-SETD2-TKO_ChIPseq_H3K4me3_rep3_batch2: 54604904
C3H10T1-2_NSD1-2-3-SETD2-QKO_ChIPseq_H3K4me3_rep1_batch2: 45420955
C3H10T1-2_NSD1-2-3-SETD2-QKO_ChIPseq_H3K4me3_rep2_batch2: 46997615
C3H10T1-2_NSD1-2-3-SETD2-QKO_ChIPseq_H3K4me3_rep3_batch2: 52447297
C3H10T1-2_PA_SUV39H1_FLAG : 21869901
C3H10T1-2_TKO_SUV39H1_FLAG : 31234868
C3H10T1-2_PA_HP1 : 51038166
C3H10T1-2_TKO_HP1 : 61236216
C3H10T1-2_PA_SUV39H1_FLAG_input : 40779593
C3H10T1-2_TKO_SUV39H1_FLAG_input : 42461128
C3H10T1-2_PA_HP1_input : 32811899
C3H10T1-2_TKO_HP1_input : 25733135
HNSCC_Cal27_H3K36M_ChIPseq_input: 30749335
HNSCC_Cal27_Parental_sgCtrl_ChIPseq_input: 12846417
HNSCC_Cal27_H3K36M_ChIPseq_H3K9me3: 39534880
HNSCC_Cal27_Parental_sgCtrl_ChIPseq_H3K9me3: 36922491
HNSCC_Cal27_H3K36M_OE_input_batch2: 41331251
HNSCC_Cal27_Parental_ChIPseq_input_batch2: 40607292
HNSCC_Cal27_Parental_ChIPseq_H3K27me3_batch2: 40035839
HNSCC_Cal27_H3K36M-OE_ChIPseq_H3K27me3_batch2: 42360704
HNSCC_Cal27_Parental_ChIPseq_H3K27ac_rep1_batch2: 53681913
HNSCC_Cal27_Parental_ChIPseq_H3K27ac_rep2_batch2: 72237870

HNSCC_Cal27_H3K36M_OE_ChIPseq_H3K27ac_rep1_batch2: 39828373
HNSCC_Cal27_H3K36M_OE_ChIPseq_H3K27ac_rep2_batch2: 88598925
HNSCC_Detroit562_Parental_sgCtrl_ChIPseq_input: 26120358
HNSCC_Detroit562_Parental_ChIPseq_input : 22713709
HNSCC_Detroit562_H3K36M_OE_ChIPseq_input: 27454245
HNSCC_Detroit562_H3K36M_OE_ChIPseq_H3K9me3: 38058856
HNSCC_Detroit562_Parental_sgCtrl_ChIPseq_H3K27me3: 34526423
HNSCC_Detroit562_H3K36M_OE_ChIPseq_H3K27me3: 56443036
HNSCC_Detroit562_Parental_sgCtrl_ChIPseq_H3K27ac: 52672255
HNSCC_Detroit562_Parental_ChIPseq_H3K27ac : 46510405
HNSCC_Detroit562_H3K36M_OE_ChIPseq_H3K27ac_rep1: 60003800
HNSCC_Detroit562_H3K36M_OE_ChIPseq_H3K27ac_rep2: 65336050

Antibodies

For ChIP-seq and CUT&RUN the following primary antibodies were used: anti-H3K36me2 (CST, 2901), anti-H3K27ac (Diagenode, C15410196), anti-H3K4me1 (CST, 5326S), anti-H3K4me3 (Diagenode, C15410003-50), anti-H3K27me1 (Active Motif, 61016), anti-H3K27me2 (CST, 9728), anti-H3K27me3 (CST, 9733), anti-H3K9me3 (Abcam, ab8898), anti-FLAG M2 (Sigma-Aldrich, F3165), anti-HP1 (CST, 2616)

Peak calling parameters

Peak calling for the C3H10T1/2 NSD1/2-SETD2-TKO samples was performed using Epic2 (v.0.0.52) with the parameters '--genome mm10 --fdr 0.01', using the corresponding input files as controls.

Data quality

ChIP-sequencing experiments were evaluated based on the percentage of reads mapped to mm10/hg38 and, when applicable, to the dm6 genome, to ensure adequate coverage. Reads with low mapping quality were excluded from downstream analyses. Antibody pulldown efficiency was visually assessed by inspecting signal tracks and comparing them to the distribution of spiked-in reference marks, when applicable.

Software

Raw ChIP-Seq reads were aligned to a combined reference of mm10 (or to hg38 for HNSCC cells) and dm6 genome assembly using BWA version 0.7.17 with default parameters. Afterwards, they were filtered using a cut-off of MAPQ < 3 using Samtools v.1.18. Samclip v.0.2 was used to filter any bacterial sequences. Read counting in bins was performed using BEDTools v.2.22.1. ChIP-seq coverage tracks were visualized using IGV v2.3 and plotted using pyGenomeTracks v.3.2.1. bigWig files were generated using the bamCoverage function from deepTools v3.3.1 with parameters '-b ${samp}.sorted.bam -o ${samp}.cpm.bw -p $nprocs --normalizeUsing CPM --centerReads -e 200 -bs 10'. Heatmaps and aggregate (average) profiles of bigwig pileups at specific genomic regions, such as accessible enhancers and promoters, were generated using the computeMatrix, plotProfile and plotHeatmap functions from deepTools78 v3.3.1. Replicates (n=2 or n=3) were merged prior to plotting, when applicable, using bigwigCompare from deepTools with parameters ‘-b1 rep1 -b2 rep2 $outdir --operation mean -bs 10 -o $merged.cpm.bw’. Strong enhancers were computed from ChIP-seq H3K27ac using ROSE v1.0.0 with parameters “-s 12500 -t 2500”. Genome-wide Pearson’s correlation analysis was performed using the multiBigWigSummary function from DeepTools using 500 bp bins and subsequently plotted using gplots v3.1.3. ChIPbinner v.0.99.1 was used to generate scatterplots of genic/intergenic regions and density-based clusters for ChIP-seq signal binned in uniform 10 or 100kb windows. plotEnrichment from deepTools was used to compute the percentage of read counts found in cluster A and B regions. Profileplyr v1.6.0 was used to extract and summarize signal values from DeepTools’ computeMatrix output for visualization with ggplot2 and statistical comparison across cluster A and B regions. Custom R scripts used to generate plots of "peakiness scores" as well as all other plots can be found at: https://github.com/padilr1/H3K36me_guardian_epigenome_integrity_Padilla.git
